# Result of one-year, prospective follow-up of intensive care unit survivors after SARS-CoV-2 pneumonia

**DOI:** 10.1186/s13613-022-00997-8

**Published:** 2022-03-09

**Authors:** Guillaume Eberst, Fréderic Claudé, Lucie Laurent, Aurelia Meurisse, Pauline Roux-Claudé, Cindy Barnig, Dewi Vernerey, Sophie Paget-Bailly, Kevin Bouiller, Catherine Chirouze, Julien Behr, Franck Grillet, Ophélie Ritter, Sinan Karaer, Sébastien Pili-Floury, Hadrien Winiszewski, Emmanuel Samain, Pierre Decavel, Gilles Capellier, Virginie Westeel

**Affiliations:** 1 Respiratory Medicine Department, University Hospital of Besançon, 3 Boulevard Fleming, 25030 Besançon, France; 2 Methodology and Quality of Life in Oncology Unit, University Hospital, Besançon, France; 3 UMR 1098, University of Franche-Comté, Besançon, France; 4 Department of Infectious Disease, University Hospital of Besançon, Besançon, France; 5 Department of Radiology, University Hospital of Besançon, Besançon, France; 6 Anesthesia and Intensive Care Unit, University Hospital of Besançon, Besançon, France; 7 Medical Intensive Care Unit, University Hospital of Besançon, Besançon, France; 8 Research Unit EA3920, Université de Franche Comté, Besançon, France; 9 Laboratory of Clinical Functional Exploration of Movement, Department of Physical Medicine and Rehabilitation, University Hospital of Besançon, Besançon, France; 10 Australian and New Zealand Intensive Care Research Center, Department of Epidemiology and Preventive Medicine, Monash University, Melbourne, Australia

**Keywords:** SARS-CoV-2 pneumonia, Acute respiratory distress syndrome, Pulmonary functional outcomes

## Abstract

**Introduction:**

Survivors of viral ARDS are at risk of long-term physical, functional and neuropsychological complications resulting from the lung injury itself, but also from potential multiorgan dysfunction, and the long stay in the intensive care unit (ICU). Recovery profiles after severe SARS-CoV-2 pneumonia in intensive care unit survivors have yet to be clearly defined.

**Material and methods:**

The goal of this single-center, prospective, observational study was to systematically evaluate pulmonary and extrapulmonary function at 12 months after a stay in the ICU, in a prospectively identified cohort of patients who survived SARS-CoV-2 pneumonia. Eligible patients were assessed at 3, 6 and 12 months after onset of SARS-CoV-2. Patients underwent physical examination, pulmonary function testing, chest computed tomography (CT) scan, a standardized six-minute walk test with continuous oximetry, overnight home respiratory polygraphy and have completed quality of life questionnaire. The primary endpoint was alteration of the alveolar–capillary barrier compared to reference values as measured by DLCO, at 12 months after onset of SARS-CoV-2 symptoms.

**Results:**

In total, 85 patients (median age 68.4 years, (interquartile range [IQR] = 60.1–72.9 years), 78.8% male) participated in the trial. The median length of hospital stay was 44 days (IQR: 20–60) including 17 days in ICU (IQR: 11–26). Pulmonary function tests were completed at 3 months (*n* = 85), 6 months (*n* = 80), and 12 months (*n* = 73) after onset of symptoms. Most patients showed an improvement in DLCO at each timepoint (3, 6, and 12 months). All patients who normalized their DLCO did not subsequently deteriorate, except one. Chest CT scans were abnormal in 77 patients (96.3%) at 3 months and although the proportion was the same at 12 months, but patterns have changed.

**Conclusion:**

We report the results of a comprehensive evaluation of 85 patients admitted to the ICU for SARS-CoV-2, at one-year follow-up after symptom onset. We show that most patients had an improvement in DLCO at each timepoint.

*Trial registration:* Clinical trial registration number: NCT04519320.

**Supplementary Information:**

The online version contains supplementary material available at 10.1186/s13613-022-00997-8.

## Key Points

The most interesting findings were that most patients showed an improvement in their DLCO at 3, 6, and 12 months, and all patients but one who normalized their DLCO did not deteriorate afterwards. Only 11% of patients had persistent impairment of DLCO at 1 year.

**Supplementary Information:**

The online version contains supplementary material available at 10.1186/s13613-022-00997-8.

## Introduction

In late December 2019, an outbreak of pneumonia started in Wuhan, China, caused by a novel coronavirus, which was named severe acute respiratory syndrome coronavirus 2 (SARS-CoV-2) [1], due to the occurrence of severe acute respiratory distress syndrome (ARDS) in 29% of hospitalized patients.

Although data to accurately estimate the extent of post-SARS-CoV-2 sequelae are lacking, survivors of viral ARDS are at risk of long-term physical, functional and neuropsychological complications resulting from the lung injury itself, but also from potential multiorgan dysfunction, and the long stay in the intensive care unit (ICU) [2–4]. Post-viral syndromes are well documented following other viral infections, including previous coronavirus outbreaks such as severe acute respiratory syndrome (SARS) and Middle East respiratory syndrome (MERS). SARS resulted in significant repercussions on pulmonary function, chronic musculoskeletal pain, and long-term mental disorders in survivors [5]. Chen et al. followed up 56 patients with H7N9 avian influenza to analyze pulmonary function and imaging changes up to 2 years after infection. Their results showed that despite interstitial changes and fibrosis on imaging, ventilation and diffusion dysfunction improved during the first 3 months and the improvement was associated with the sequelae observed at 2 years [6]. Wu et al. recently reported serial pulmonary function, exercise capacity, and chest high-resolution computed tomography (HRCT) changes in non-intubated patients hospitalized in Wuhan with severe SARS-CoV-2 pneumonia at 3, 6, 9, and 12 months following hospital discharge. They found evidence of persistent physiological and radiographic changes in a subgroup of patients [7]. To the best of our knowledge, there is no European report focusing exclusively on the most severe patients, as defined by the World Health Organization (WHO) categories. The long-term effect of SARS-CoV-2 on lung parenchyma and pulmonary function remains an open question.

With 295 patients hospitalized in the ICU at the peak of the epidemic in April, the region of Bourgogne-Franche-Comté in Eastern France was one of the regions with the highest incidence rates and ICU admissions for SARS-CoV-2 in France [8]. The goal of this study was to describe one-year recovery profiles, defined by repeated respiratory and exercise function, and quality of life evaluations, in a prospectively identified cohort of ICU patients who survived severe pneumonia.

## Methods

### Patients and study design of COV-RECUP

This single-center, prospective, observational study was performed in the French University Hospital of Besançon from April 2020 to June 2021 (first wave). All SARS-CoV-2 ICU survivors were contacted upon discharge from ICU and invited to participate in the trial. Patients were eligible if they had SARS-CoV-2 infection diagnosed by viral RNA detection by quantitative RT-PCR on nasal swabs or bronchoalveolar lavage. Patients had to have been admitted to the ICU with SpO_2_ < 92% and evidence of air-space changes in 25% of lung parenchyma on chest CT scan. For fear of non-compliance with follow-up procedures due to increased morbidity–mortality, patients were excluded if they were older than 79 years. Other exclusion criteria were the following: chronic respiratory insufficiency, long-term oxygen therapy, interstitial lung disease, significant psychiatric disorders, or a life expectancy estimated at less than one year. The study consisted in follow-up visits, including outpatient evaluation at 3, 6 and 12 months after symptom onset. Only for patients with sequelae, annual follow-up was planned up to a maximum of 5 years. After discharge from the ICU, all inpatients underwent targeted exercise rehabilitation twice daily for at least 20 min with a physiotherapist. Exercise rehabilitation consisted of passive range of motion, active range of motion, electrical muscle stimulation, sitting, tilting, standing, ambulation, and other mobilization techniques depending on the patient’s condition. Cardiopulmonary rehabilitation was performed with aerobic physical activity. All patients included in the study were systematically offered early psychological follow-up.

Written consent was obtained before the first visit at 3 months and the protocol was approved by the ethics committee (Comité de Protection des Personnes (CPP) Grand-Est) on 21/04/2020. The COV-RECUP study was performed in accordance with the Declaration of Helsinki and Good Clinical Practice guidelines (Clinical trial registration number: NCT04519320).

### Follow-up procedures

At 3, 6 and 12 months (± 3 weeks) after onset of SARS-CoV-2 symptoms, patients underwent a physical examination, pulmonary function testing, blood gas analysis, non-contrast enhanced chest millimeter section CT scan, resting oximetry, and a standardized six-minute walk test (6MWT) with continuous oximetry. At 3 months only, because of risk factors common to severe COVID-19 infection and sleep apnea syndrome, a complete overnight home respiratory polygraphy was performed to evaluate the frequency of sleep apnea syndrome. Routine spirometry, and single breath hemoglobin-adjusted DLCO were performed using Global Lung Function Initiative reference values [9, 10]. Maximum expiratory (MEP) and inspiratory pressures (MIP) (Platinum Elite; MGC Diagnostics Corporation, Saint Paul, Minnesota, USA) were performed using healthy subjects reference values [11]. For single breath hemoglobin-adjusted DLCO measurements, patients were instructed to hold their breath for 10 s followed by a complete and consistent exhalation, at which time an alveolar sample of exhaled gas was analyzed for calculation of uptake of CO. Six-minute walk distance (6MWD) was assessed according to established guidelines. Symptom-limited incremental cardiopulmonary exercise testing (CPET) was performed on an electronically braked cycle ergometer (Ergometrics 900, Ergoline, Bitz, Germany) and physiological data were obtained breath by breath (MGC-CPX System; MGC Diagnostics).

Chest CT scans were performed on a Revolution CT (GE Healthcare, Milwaukee, WI, USA). Imaging results were reviewed by two chest radiologists (J.B. and F.G. with, respectively, 11 and 6 years of experience) (Carestream Health, Rochester, NY, USA). Readers were blinded to the patient’s status, clinical and biological features. Readers were asked to assess presence or absence of abnormalities. The extent of lesions was graded from 0 to 4 as follows: 0 = 0%, 1 = [1–24%], 2 = [25–49%], 3 = [50–74%], 4 = [75–100%] of whole lung surface. The topography of each lesion was also assessed.

Patients were asked to complete the Hospital Anxiety and Depression Scale (HADS), to assess symptoms of anxiety and depression; and the Medical Outcomes Study 36-item Short-Form general health survey (SF-36), which measures health-related quality of life (HRQoL). The SF-36 includes eight multiple-item scales that assess physical functioning, social functioning, role physical, role emotional, mental health, pain, vitality, and general health. Scores for each dimension range from 0 (worst) to 100 (best) [12]. The HADS was developed to detect states of depression and anxiety in adults aged 16–65 years [13]. It contains an anxiety subscale (HADS-A) and a depression subscale (HADS-D), each consisting of 7 items, rated on a four-point Likert scale (0–3). A maximum count of 21 points per subscale is possible. A score of 0–7 is considered as normal, 8–10 as a borderline case, and 11–21 as a case (anxiety or depression). The questionnaire is designed to assess the participants' state over the past 2 weeks.

### Statistical analysis

Our working hypothesis was that we would observe persistent pulmonary function changes in a subgroup of patients. To describe the recovery profile of the patients at 12 months, a primary endpoint of respiratory function was chosen, namely the hemoglobin-adjusted DLCO compared to reference values. This measure provides a standardized, objective, integrated assessment of the capillary alveolus barrier and therefore pulmonary function. The primary endpoint was alteration of the alveolo-capillary barrier at 12 months after symptom onset as measured by DLCO. Continuous variables are expressed as median (interquartile range) and were compared using Mann–Whitney *U* test or Wilcoxon signed rank test; categorical variables are expressed as number (percentage) and were compared using chi square or Fisher’s exact test or McNemar's test, as appropriate. Comparison of parameters over time was analyzed using linear mixed models in case of a linear evolution. Unconditional logistic regression models were performed to estimate the odds ratio (OR) and 95% confidence interval (CI) for factors associated with altered DLCO at 3 months. The relation between baseline clinical and biological parameters and altered DLCO was first assessed by univariate analyses. Continuous variables were transformed into categorical variables using the median, tertiles and receiving operating characteristic (ROC) curves to identify the best cutoff. Collinearity among variables was assessed using a correlation matrix. For variables with significant correlations (defined by a correlation coefficient ≥ 0.3 associated with a *p*-value < 0.001), only one variable was selected for inclusion in the multivariable model. The Concato rule (1 variable for at least 10 events) was applied. The most relevant clinical variables with a *p*-value < 0.05 by univariate analysis were selected for inclusion in the multivariate analysis.

All analyses were performed using SAS version 9.4 (SAS Institute, Cary NC) and R (version 4.0.5). *p* values of less than 0.05 were considered statistically significant, and all tests were two-sided. No adjustment was performed for multiple testing.

## Results

### Characteristics of the study population at ICU admission

**Fig. 1:**
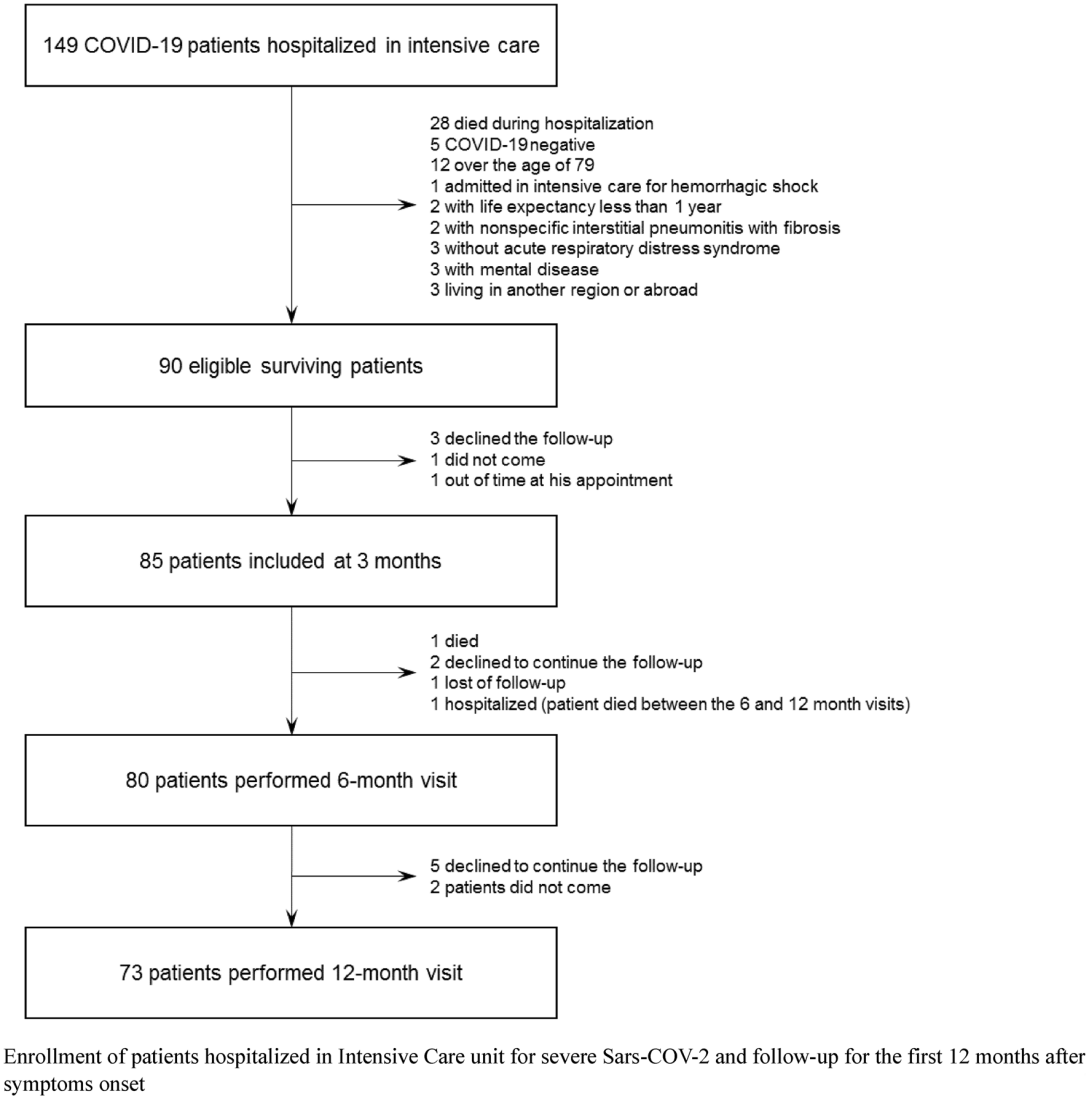
Flowchart of patients with COVID-19. Enrollment of patients hospitalized in intensive care unit for severe Sars-COV-2 and follow-up for the first 12 months after symptoms onset

A total of 149 patients with an initial diagnosis of severe SARS-CoV-2 pneumonia were admitted to the ICU; among these, 90 were eligible for the study and 85 participated (Fig. 1). Fifty-nine patients were ineligible for the following reasons: 28 deaths, 5 negatives for SARS-CoV-2 (negative quantitative RT-PCR), 12 were aged > 79 years, and 14 for other reasons (Fig. 1). Among the 90 eligible patients, 3 refused to participate, 1 did not show up for the appointment and 1 patient was outside the time limits for inclusion. The median follow-up for the full cohort was 12 months (interquartile range (IQR): 11.5–12.3). Twelve patients were lost to follow-up, 5 between 3 and 6 months (1 died, 2 declined to continue follow-up, 1 lost to follow-up and 1 hospitalized for intercurrent disease), and 7 between 6 and 12 months (5 declined to continue follow-up and 2 patients did not show up for their appointment).

The demographic characteristics of study population (*N* = 85) are detailed in Table 1. Median age was 68.4 years (IQR: 60.1–72.9 years). Sixty-seven patients (78.8%) were males. Only one patient was a current smoker, 57.7% were former smokers and 41.2% never-smokers. A large majority of patients had known comorbidities (92.9%), including mostly respiratory and cardiovascular disorders. Twenty-eight patients were obese (32.9%), 44 had arterial hypertension (51.8%), 21 had diabetes (24.7%) and 27 had dyslipidemia (31.8%). The median length of hospital stay was 44 days (IQR: 20–60) and the median length of stay in the ICU was 17 days (IQR: 11.0–26.5). Fifty-seven patients (67.1%) required admission to a rehabilitation facility at hospital discharge. Thirty-six patients received steroids (45.9%), 33 during hospitalization and 3 after the 3-month evaluation.

**Table 1:** Characteristics and medical history at enrollment (3 months after COVID-19 symptoms onset)

	Patients (*n* = 85)
Age, years	68.4 (60.1–72.9)
Male	67 (78.8%)
Smoking status	
Current smoker	1 (1.2%)
Former smoker	49 (57.6%)
Never smoker	35 (41.2%)
Comorbidities before SARS-CoV-2 infection	
Obesity	28 (32.9%)
Class I (BMI 30–34.9)	15 (53.6%)
Class II (BMI 35–39.9)	10 (35.7%)
Class III (BMI ≥ 40)	3 (10.7%)
Cardiovascular	
Ischemic heart disease	9 (10.6%)
Heart failure	1 (1.2%)
Atrial fibrillation	7 (8.2%)
Stroke	2 (2.4%)
Respiratory diseases	
COPD	7 (8.2%)
Asthma	6 (7.1%)
Sleep apnea	16 (18.8%)
Other cardiovascular risk factors	
Hypertension	44 (51.8%)
Diabetes	21 (24.7%)
Dyslipidemia	27 (31.8%)
Thromboembolic disease	
Deep vein thrombosis	4 (4.7%)
Pulmonary embolism	0 (0.0%)
Intensive care unit	
Length of stay (days)	17 (11.0–26.5)
Intubation	73 (85.9%)
Neuromuscular blocking agents (*N* = 72)^a^	71 (98.6%)
High flow oxygen therapy^b^	35 (41.2%)
Before intubation	14 (16.5%)
After intubation	29 (41.4%)
Non-invasive ventilation^b^	12 (14.1%)
Before intubation	1 (1.2%)
After intubation	10 (14.1%)
Prone position	62 (73.8%)
Pulmonary embolism	20 (23.5%)
Hospital stay	
Length of hospitalization (days)	45 (20–62)
In-hospital COVID-directed treatments	
Corticosteroids	36 (42.4%)
Remdisivir	5 (5.9%)
Lopinavir	9 (10.6%)
Ritonavir	10 (11.8%)
Hydroxychloroquine	54 (63.5%)
Azithromycin	45 (52.9%)
Others macrolides	55 (64.7%)
Rehabilitation post hospitalization	
Admission in rehabilitation units	57 (67.1%)
Return home with rehabilitation	11 (12.9%)
Return home without rehabilitation	17 (20.0%)

Values are presented as medians (interquartile ranges) or number of patients (percentages)

*BMI* body mass index, *COPD* chronic obstructive pulmonary disease

^a^Data were unavailable for 1 the 73 intubate patients

^b^Details were missing for 2 patients in high-flow oxygen therapy and 1 for non-invasive ventilation

### Recovery profiles

Pulmonary function tests were completed in all patients at 3 months (*n* = 85), in 80 patients at 6 months, and in 73 patients at 12 months following onset of SARS-CoV-2. Most patients showed an improvement in their DLCO at each timepoint and patients who normalized their DLCO did not subsequently deteriorate. Forty-nine patients had returned to normal DLCO at 3 months (58%), 66 (85%) at 6 months, and 63 (89%) at 12 months (Additional file 1: Fig. S1). Median DLCO was 80% of predicted (IQR 64–91) at 3 months, 91% of predicted (79–104) at 6 months, and increased to 98% of predicted (88–107) at 12 months (*p* < 0.0001). Eight patients (11%) presented a DLCO below the lower limit of normal (LLN) at 12 months. Among patients with a DLCO below the LLN at 12 months, a mild-to-moderate reduction in DLCO was observed, with median DLCO at 62.0% of predicted (IQR 52.0 to 69.1 percent of the predicted values) (Table 2).

**Table 2:** Functional, physiological and CT scan data at 3, 6 and 12 months

	3 months (*N* = 85)	6 months (*N* = 80)	12 months (*N* = 73)	*p*-value M3–M6	*p*-value M6–M12	*p*-value Mixed model
Dyspnea (mMRC scale)						
Number evaluated	83 (97.6%)^a^	78 (97.5%)^a^	73 (100%)			
0	30 (36.1%)	34 (43.6%)	35 (47.9%)	0.3397	0.1951	NA
1	39 (47.0%)	32 (41.0%)	34 (46.6%)			
2	8 (9.6%)	12 (15.4%)	14 (5.5%)			
3–4	6 (7.2%)	0 (0.0%)	0 (0.0%)			
Oxygen therapy						
Ambulatory oxygen therapy	5 (5.9%)	4 (5.0%)	4 (5.5%)	–	–	NA
Long-term oxygen therapy	3 (3.5%)	1 (1.3%)	1 (1.4%)	0.3173	1	NA
Lung volumes, spirometry and diffusing capacity						
FVC (% of pred)	92.0 (80.0–103.6)	99.0 (86.6–112.1)	105.0 (92.0–120.0)	** < 0.0001**	** < 0.0001**	** < 0.0001**
Altered FVC (*Z* score FVC < − 1.64)	18 (21.2%)	8 (10.0%)	4 (5.5%)	**0.0067**	0.1797	NA
FEV1 (% of pred)	92.0 (81.0–103.2)	99.7 (88.4–115.0)	104.0 (92.0–118.0)	** < 0.0001**	** < 0.0001**	** < 0.0001**
FEV1/FVC (%)	82.0 (74.0–86.0)	81.5 (74.5–86.0)	81.0 (73.0–85.0)	**0.0267**	**0.0033**	**0.0275**
FEV1/FVC < 70%	12 (14.1%)	10 (12.5%)	12 (16.4%)	0.5637	0.3173	NA
DLCO (% of pred)	80.0 (64.0–91.2)	90.9 (79.9–103.8)	98.3 (88.4–106.5)	** < 0.0001**	** < 0.0001**	** < 0.0001**
Altered DLCO (*Z* score DLCO < − 1.64)	36 (42.4%)	12 (15.4%)	8 (11.0%)	** < 0.0001**	0.3173	NA
KCO (% of pred)	88.8 (76.5–104.0)	98.3 (86.0–109.5)	102.5 (94.3–114.4)	** < 0.0001**	** < 0.0001**	** < 0.0001**
Respiratory muscle strength						
MIP (% of pred)	74.5 (54–95)	89 (70–105)	99 (77–122)	** < 0.0001**	**0.0002**	** < 0.0001**
MEP (% of pred)	50 (40–59)	54 (45–68)	57 (48–69)	**0.0095**	0.2343	**0.0079**
SNIP (% of pred)	84 (68–99)	87 (73–102)	94 (82–120)^b^	**0.0376**	** < 0.0001**	** < 0.0001**
FVC in supine position (% of pred)	92 (76.5–104)	98 (84–108)	100 (86.5–112.5)§	** < 0.0001**	**0.0003**	** < 0.0001**
Change in FVC in supine position (%)	− 4 ((− 7)–(− 1.5))	− 4 ((− 8)–(− 2))	− 4 ((− 8)–(− 2))^c^	0.4697	0.5732	0.4534
Blood gas						
PaO_2_ (kPa)	11.1 (10.2–12.3)	–	10.7 (10.1–11.6)	–	–	0.0784
PaO_2_ < 9.3 kPa	11 (12.9%)	–	5 (6.8%)	–	–	NA
PaCO_2_ (kPa)	4.8 (4.5–5.1)	–	4.9 (4.6–5.2)	–	–	0.1140
6MWT						
Number evaluated	79 (92.9%)^d^	76 (95.0%)^f^	66 (90.4%)ǁ	–	–	
Walked distance (m)	481 (400–564)	549.5 (472.5–600.0)	542 (495–600)	** < 0.0001**	0.6211	** < 0.0001**
Walked distance < theoretical distance (%)	50 (63.3%)	26 (34.2%)	18 (27.3%)	** < 0.0001**	0.3938	NA
Loss of 4% or more of SpO_2_	45 (57.0%)	32 (42.1%)	25 (37.9%)	0.1172	0.2971	NA
SpO_2_ < 88% at the end of the test	9 (11.4%)	5 (6.6%)	4 (6.1%)	0.1797	1	NA
Dyspnea on Borg scale before exercise	0 (0–2)	0 (0–1)	0.5 (0–2)	**0.0323**	**0.0025**	0.4059
Dyspnea on Borg scale after exercise	4 (2–6)	4 (3–6)	4 (3–6)	0.2528	0.4482	0.2724
Fatigue on Borg scale before exercise	0 (0–3)	0 (0–1)	0 (0–2)	**0.0010**	0.3471	0.2414
Fatigue on Borg scale after exercise	3 (1–5)	3 (0–3.5)	2 (0–4)	**0.0004**	0.7865	0.0646
Respiratory polygraphy						
Number evaluated	68 (80.0%)^g^	–	–	–	–	
Sleep apnea syndrome (AHI ≥ 5)	62 (91.2%)					
Central events (%)	5.2 (0.6–17.9)	–	–	–	–	
Hospital Anxiety and Depression Scale						
Anxiety ≥ 11	7 (8.2%)	7 (8.8%)	7 (9.6%)	0.7055	1	NA
Depression ≥ 11	3 (3.5%)	4 (5.0%)	3 (4.1%)	0.6547	1	NA
CT scan						
Number evaluated	80 (94.1%)^h^	–	64 (90.1%)^i^	–	–	
Abnormal scan	77 (96.3%)	–	60 (93.8%)	–	–	
Reticulations	69 (89.6%)	–	51 (85.0%)	–	–	
1–25%	52 (75.4%)	–	42 (82.4%)	–	–	
26–50%	14 (20.3%)	–	9 (17.6%)	–	–	
51–75%	3 (4.3%)	–	0 (0.0%)	–	–	
Traction bronchiectases	53 (68.8%)	–	44 (73.3%)	–	–	
1–25%	43 (81.1%)	–	42 (95.5%)	–	–	
26–50%	10 (18.9%)	–	2 (4.5%)	–	–	
Honeycombing	6 (7.8%)	–	3 (5.0%)	–	–	
1–25%	4 (66.7%)	–	3 (100%)	–	–	
26–50%	2 (33.3%)	–	0 (0.0%)	–	–	
Ground-glass opacities	56 (72.7%)	–	32 (53.3%)	–	–	
1–25%	42 (75.0%)	–	30 (93.8%)	–	–	
26–50%	10 (17.9%)	–	1 (3.1%)	–	–	
51–75%	4 (7.1%)	–	1 (3.1%)	–	–	
Emphysema	14 (18.2%)	–	12 (20.0%)	–	–	
1–25%	6 (42.9%)	–	5 (41.7%)	–	–	
26–50%	3 (21.4%)	–	3 (25.0%)	–	–	
51–75%	1 (7.1%)	–	2 (16.7%)	–	–	
> 75%	4 (28.6%)	–	2 (16.7%)	–	–	
Cardiorespiratory stress test		–				
Number evaluated	–	–	61 (83.6%)^j^			
Pmax (watts)	–	–	123 (103–153)	–	–	
VO_2_ peak (% of pred)	–	–	99 (88–106)	–	–	

Values are presented as medians (interquartile ranges) or number of patients (percentages)

*ICU* intensive care unit, *FVC* forced vital capacity, *FEV1*  forced expiratory volume in 1 s, *DLCO*  diffusing capacity of the lung for carbon monoxide, *FeNO*  fractional exhaled nitric oxide, *MIP*  maximal inspiratory pressure, *MEP* maximal expiratory pressure, *SNIP*  sniff nasal inspiratory pressure, *6MWT*  six-minute-walk test, *CT scan*  computerized tomography scan

^a^Dyspnea could not be evaluated for the two same patients at 3 and 6 months because they were not yet able to walk

^b^SNIP could not be evaluated for 27 patients for technical issue

^c^1 patient felt faint with head trauma during respiratory evaluation and was unable to continue the examinations

^d^6 patients were not able to perform the 6MWT at 3 months

^e^4 patients were not able to perform the 6MWT at 6 months

^f^7 patients were not able to perform the 6MWT at 12 months

^g^Polygraph was not available for 11 patients and 6 patients had inoperable polygraphs

^h^5 patients could not get a CT scan for technical issue

^i^8 patients had a Chest-X Ray and 1 patient could not get a CT scan for technical issue

^j^12 patients were not able to perform the cardiorespiratory stress test at 12 months

On the 6MWT, 50 patients (63.3%) had walk distances below their age-adjusted predicted values at 3 months (overall median walk distance = 481 m (IQR: 400–564 m)), and 18 (27.3%) at 12 months (overall median walk distance = 542 m (IQR: 495–600 m)) (*p* < 0.0001). The proportion of patients whose arterial oxygen saturation fell below 88 percent during the 6MWT was 11.4% at 3 months and 6.1% at 12 months. VO_2_ peak in incremental CPET was a median of 99% of predicted at 12 months (IQR: 88–106). Sixty-eight patients had an overnight polygraphy recording. Sixty-two patients presented on obstructive sleep apnea syndrome with an apnea–hypopnea index (AHI) ≥ 5 (91.2) (Table 2).

Chest CT scans were abnormal in 77 patients (96.3%) at 3 months. When present, radiologic changes included ground glass opacities in most cases (72.7%), atelectases, nodules and alveolar consolidations. At 12 months, the proportion of abnormal CT scan was the same, but there was a change in the patterns (96% vs 95% were abnormal CT Scan). The proportion of scans with ground glass dropped (73% to 53%), but there were more reticulations (10% vs 86%) and traction bronchiectases (69% vs 72%). The extent of each type of lesion decreased during follow-up (Table 2).

### Quality of life assessment

**Fig. 2:**
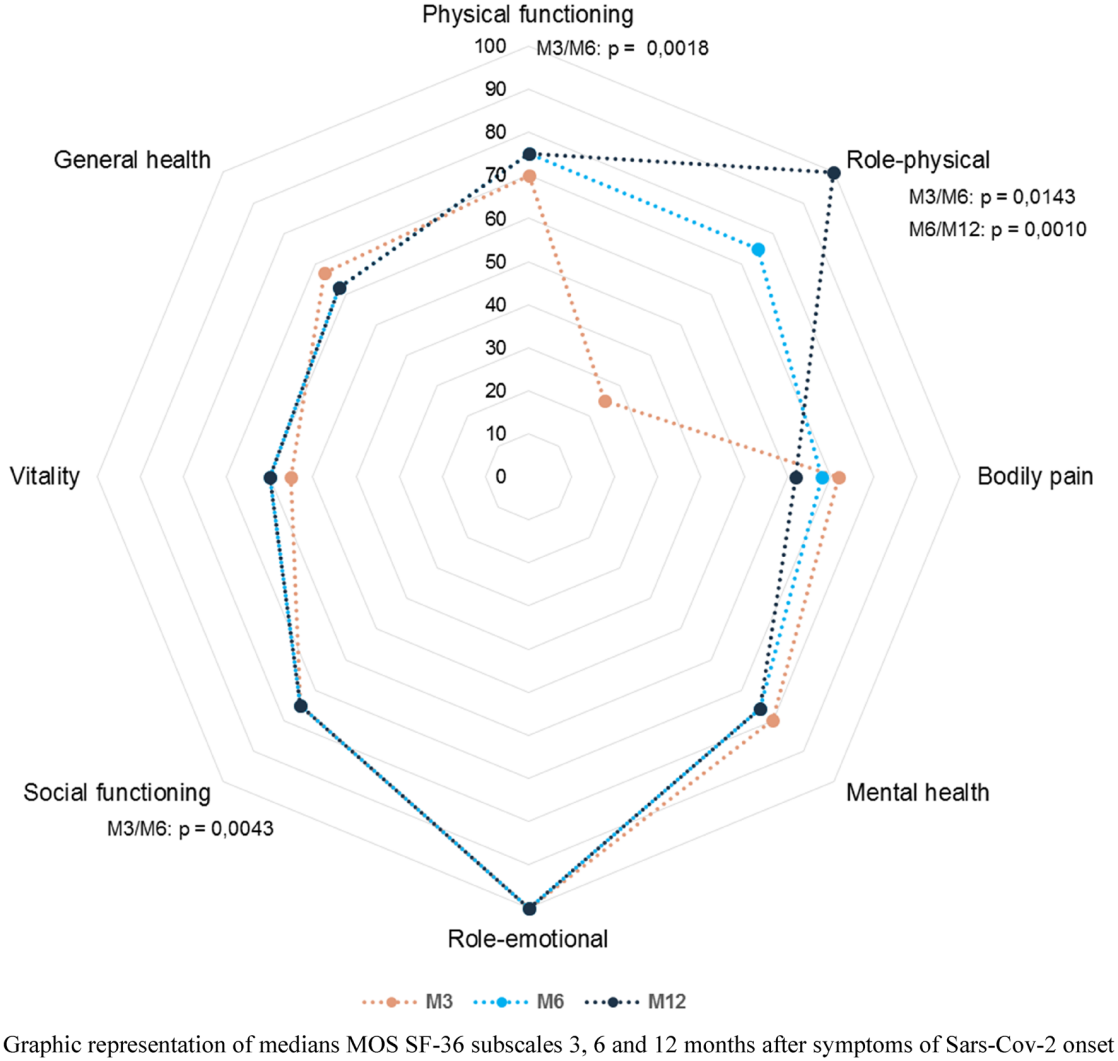
Results of Quality of Life Assessments at 3, 6 and 12 months using the Medical Outcomes Study Short Form 36-item questionnaire. Graphic representation of medians MOS SF-36 subscales 3, 6 and 12 months after symptoms of Sars-Cov-2 onset

**Fig. 3:**
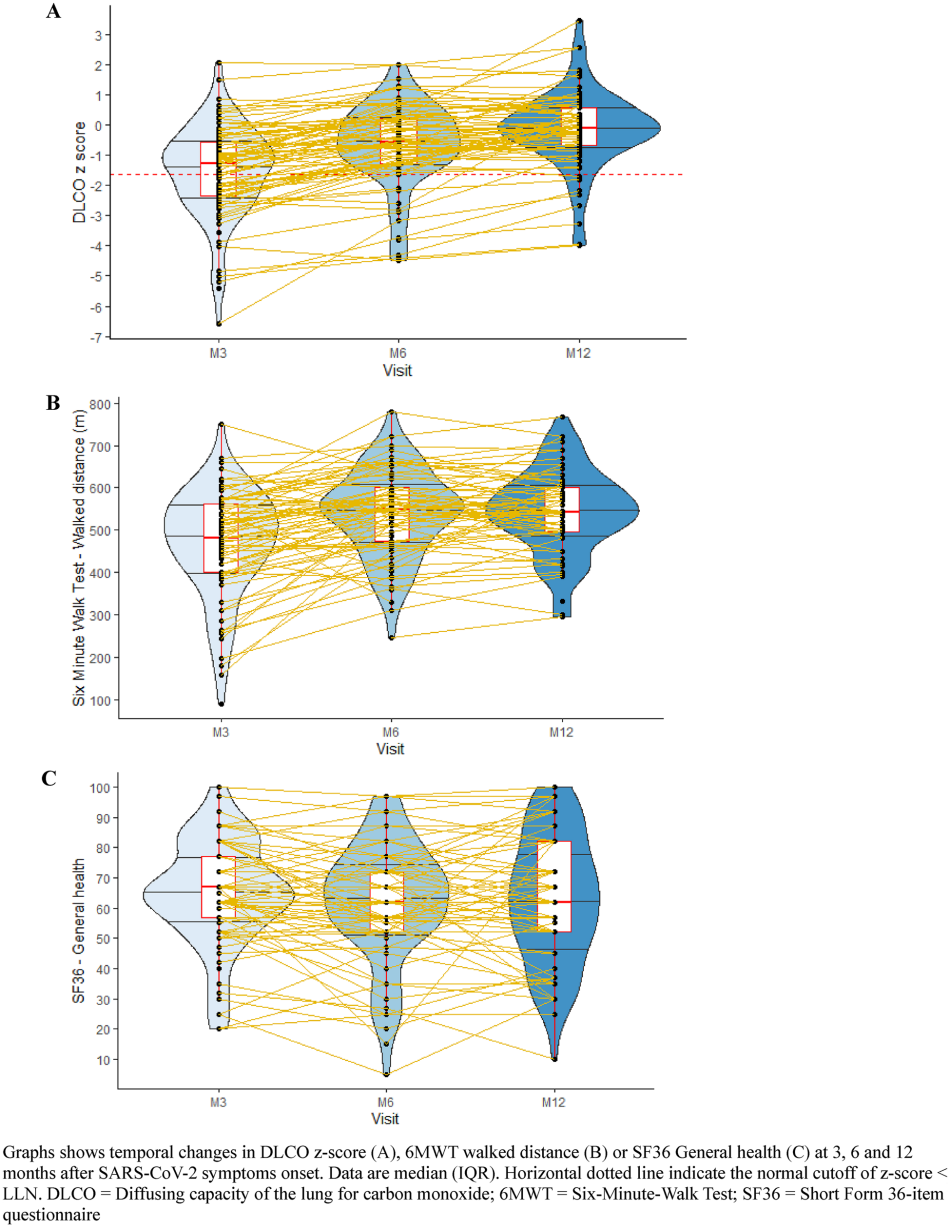
Temporal changes in pulmonary function of ICU survivors of severe SARS-CoV-2 infection at 3, 6 and 12 months after symptoms onset. Graphs shows temporal changes in DLCO z-score (**A**), 6MWT walked distance (**B**) or SF36 General health (**C**) at 3, 6 and 12 months after SARS-CoV-2 symptoms onset. Data are median (IQR). Horizontal dotted line indicate the normal cutoff of z-score < LLN. *DLCO* diffusing capacity of the lung for carbon monoxide; *6MWT*  six-minute-walk test; *SF36* Short Form 36-item questionnaire

All 85 patients completed the SF-36 questionnaire at 3 months. The scores for all domains of the SF-36 remained slightly below 100, three months after the first symptoms of SARS-CoV-2 except for one, namely impairment of work or other regular daily activities as a result of any emotional problem, where scores were normal (Fig. 2). The two domains with the lowest scores were vitality (55) and role-physical (25). In the 18 patients (27.7%) who had walk distances below their age-adjusted predicted values on the 6MWT at 3 months, the median in the vitality domain was 55 (IQR: 45–80). Most patients showed an improvement in their quality of life assessments at each timepoint (3, 6, and 12 months). The domain with the greatest improvement was the role-physical scale, which increased from 25 to 100 (Fig. 3).

Regarding the evaluation using the HADS, 3 patients were found to have symptoms of depression (3.5%) and 7 had symptoms of anxiety (8.2%).

### Association with DLCO impairment

Table 3 presents the follow-up parameters and their relation with altered DLCO at 3, 6, 12 months. At 12 months, emphysema on CT scan was significantly associated with altered DLCO. Walked distance < theoretical distance (%) on the 6MWT was associated with altered DLCO at 3 and 6 months. No association was found between patient-reported outcomes and DLCO alteration (Table 3). For 6 of the 8 patients with an alteration of their DLCO, we concluded they had emphysema, which presumably existed before SARS-CoV-2. For one patient, after checking previous CT scans, we concluded that there had been a flare of pre-existing undiagnosed interstitial lung disease concurrent with the SARS-CoV-2 infection. For the last patient, there was persistent interstitial lung disease at 12 months, but the patient had no CT scans dating from before SARS-Cov-2 that could be used for comparison.

**Table 3:** Follow-up parameters associated with DLCO alteration at 3 and 12 months

	3 months			12 months		
	Normal DLCO *N* = 49	Altered DLCO *N* = 36	*p*-value	Normal DLCO *N* = 65	Altered DLCO *N* = 8	*p*-value
6MWT						
Number evaluated	46 (93.9%)	33 (91.7%)	0.6948	59 (90.8%)	7 (87.5%)	0.5727
Walked distance (m)	510 (450–570)	443 (370–525)	**0.0199**	540 (500–612)	545 (420–590)	0.4620
Walked distance < theoretical distance (%)	23 (50.0%)	27 (81.8%)	**0.0038**	15 (25.4%)	3 (42.9%)	0.3800
Hospital Anxiety and Depression Scale						
Anxiety ≥ 11	6 (12.2%)	1 (2.8%)	0.2302	7 (10.8%)	0 (0.0%)	1
Depression ≥ 11	2 (4.1%)	1 (2.8%)	1	3 (4.6%)	0 (0.0%)	1
MOS-SF36						
Physical functioning	80 (60–90)	65 (27.5–85)	0.0651	75 (65–90)	72.5 (60–82.5)	0.4695
Role-physical	50 (0–100)	25 (0–87.5)	0.3489	100 (25–100)	75 (0–100)	0.3428
Bodily pain	62 (42–84)	72 (50.5–84)	0.6144	62 (41–84)	63 (57–87)	0.5058
Mental health	80 (60–92)	76 (64–90)	0.6822	76 (60–88)	86 (74–90)	0.3928
Role-emotional	66.7 (0–100)	100 (0–100)	0.3206	100 (33.3–100)	83.3 (33.3–100)	0.9074
Social functioning	75 (50–100)	68.8 (43.8–100)	0.5403	75 (50–100)	87.5 (81.3–100)	0.2072
Vitality	55 (45–70)	55 (47.5–72)	1	60 (45–75)	57.5 (52.5–72.5)	0.8253
General health	67 (57.82)	63.5 (54.5–72)	0.3808	67 (52–82)	58.5 (52–67)	0.5428
CT scan						
Missing data	5	0		9	0	
Abnormal scan	41 (93.2%)	36 (100%)	0.2481	56 (88.9%)	8 (100%)	1
Reticulations	37 (90.2%)	32 (88.9%)	0.8458	52 (92.9%)	6 (75.0%)	0.5928
Traction bronchiectasis	22 (53.7%)	31 (86.1%)	**0.0029**	38 (73.1%)	6 (75.0%)	1
Honeycombing	2 (4.9%)	4 (11.1%)	0.4099	3 (5.8%)	0 (0.0%)	1
Ground-glass opacity	29 (70.7%)	27 (75.0%)	0.6748	30 (57.7%)	2 (25.0%)	0.1300
Emphysema	4 (9.8%)	10 (27.8%)	**0.0408**	6 (11.5%)	6 (75.0%)	**0.0004**

Values are presented as medians (interquartile ranges) or number of patients (percentages)

*6MWT*  six-minute-walk test, *MOS-SF36*  medical outcomes study short form 36-item questionnaire, *CT scan*  computerized tomography scan

### Risk factors associated with impaired DLCO

Univariate analysis identified three factors that were significantly associated with impaired DLCO at 3 months, namely length of stay in ICU (days), creatininemia and obesity. No risk factor for altered DLCO at 6 and 12 months was identified (Tables 4, 5).

**Table 4:** Baseline characteristics associated with DLCO alteration at 3, 6 and 12 months

	3 months			6 months			12 months		
	Normal DLCO *N* = 49	Altered DLCO *N* = 36	*p*-value	Normal DLCO *N* = 66	Altered DLCO *N* = 12	*p*-value	Normal DLCO *N* = 65	Altered DLCO *N* = 8	*p*-value
Age, years	67.3 (59.7–73.3)	68.8 (62.4–72.8)	0.5440	68.6 (60.1–73.3)	67.8 (63.3–71.2)	0.6689	68.7 (60.1–72.9)	66.2 (59.4–71.4)	0.8534
Male	35 (71.4%)	32 (88.9%)	0.0516	49 (74.2%)	12 (100%)	0.0589	50 (76.9%)	7 (87.5%)	0.6760
Length of hospitalization (days)	39 (20–52)	53 (24–80)	**0.0313**	44 (20–61)	58.5 (24–102)	0.2227	45 (20–65)	60 (54–102)	0.1529
Intensive care unit									
Obesity									
Missing data	2	0		2	0		2	0	
BMI < 25	4 (8.5%)	10 (27.8%)	**0.0104**	11 (17.2%)	3 (25.0%)	0.6590	11 (17.5%)	2 (25.0%)	0.3387
BMI ≥ 25—< 30	21 (44.7%)	19 (52.8%)		28 (43.8%)	6 (50.0%)		28 (44.4%)	5 (62.5%)	
BMI ≥ 30	22 (46.8%)	7 (19.4%)		25 (39.1%)	3 (25.0%)		24 (38.1%)	1 (12.5%)	
Length of stay in ICU (days)	16.5 (11–22)	19.5 (11–37.5)	0.1016	17 (11–23)	32 (13.5–39)	**0.0274**	17.5 (11–24)	34 (12.5–39)	0.8534
Creatinemia	81.6 (68.7–98)	113.3 (84.7–163.8)	**0.0005**	84.8 (70.4–113)	125.5 (93.6–163.8)	**0.0052**	88 (71–116.4)	110 (84.6–147.6)	0.1400
CRP	184.1 (128–259)	193.5 (124.9–276)	0.7329	185.6 (128–270.7)	174.5 (108.5–244.1)	0.4864	189 (121.2–270.7)	143.4 (112.6–193.5)	0.3391
Score SOFA	3 (2–6)	3 (2–6)	0.8614	3 (2–5)	3 (2–6)	0.8464	3 (2–6)	2.5 (2–5.5)	0.6985
Neuromuscular blocking agents	40 (83.3%)	31 (86.1%)	0.7276	57 (87.7%)	10 (83.3%)	0.6503	58 (90.6%)	6 (75.0%)	0.2150
Prone position	35 (72.9%)	27 (75.0%)	0.8298	48 (73.8%)	10 (83.3%)	0.7192	51 (79.7%)	6 (75.0%)	0.6687
Corticosteroids	17 (34.7%)	19 (52.8%)	0.0955	25 (37.9%)	7 (58.3%)	0.2145	28 (43.1%)	6 (75%)	0.1346
Non-invasive ventilation support	3 (6.1%)	9 (25.0%)	**0.0135**	6 (9.1%)	5 (41.7%)	**0.0104**	7 (10.8%)	3 (37.5%)	0.0728
PaO^2^/FiO^2^ (at Day 0 of ICU)	137.5 (108.8–197.7)	157.5 (118.9–216.2)	0.5292	140 (111.4–206.9)	154.5 (85.5–225)	0.9684	153 (111.4–208.4)	166 (105.4–202.5)	0.8570

Values are presented as medians (interquartile ranges) or number of patients (percentages)

*DLCO*  diffusing capacity of the lung for carbon monoxide, *BMI* body mass index, *CRP*  C-reactive protein, *SOFA*  Sequential Organ Failure Assessment, *PaO*^*2*^*/FiO*^*2*^  partial pressure of oxygen/fraction of inspired oxygen, *ICU*  intensive care unit

**Table 5:** Predictive factors for altered DLCO at 3 months

	Univariate analysis		Multivariate analysis	
	OR (95%CI)	*p*-value	OR (95%CI)	*p*-value
Intensive care unit				
Length of stay in ICU(days)	4.998 (1.694–14.747)	**0.0036**	7.707 (2.163–27.645)	**0.0016**
Obesity		**0.0153**		**0.0086**
BMI < 25	1		1	
BMI ≥ 25—< 30	0.362 (0.0097–1.348)		0.188 (0.043–0.816)	
BMI ≥ 30	0.127 (0.0030–0.536)		0.068 (0.012–0.377)	
Creatinemia > = 92.8	5.000 (1.973–12.669)	**0.0007**	4.305 (1.461–12.683)	**0.0081**

*OR*  odds ratio, *CI*  confidence interval, *BMI*  body mass index

## Discussion

In this study, we report the results of a longitudinal evaluation of 85 patients admitted to the ICU for SARS-CoV-2, with a follow-up of one year. To the best of our knowledge, no reports have described such a large European cohort of SARS-CoV-2 survivors after ICU, with systematic comprehensive evaluation including lung function testing, 6MWT, incremental CPET, thoracic CT-Scan, polygraphy and HRQoL, with an excellent compliance of 86% at 1 year. A strength of our study is the complete longitudinal follow-up with repeated measures, making it possible to qualify the recovery of our patients as a function of time.

The most interesting findings were that only 8 patients (11%) having persistent impairment of DLCO at 1 year. Among these 8 patients, due to pre-existing, undiagnosed respiratory comorbidities in 7 patients, only 1 patient was likely to actually have COVID-19-related DLCO alteration at 1 year. The proportion of patients with abnormal DLCO in our study is lower than that reported by Wu et al., which was 32.5% [7]. This might be explained by the use of different reference values compared than in Wu’s cohort who used the American Thoracic Society guidelines of 1994 [14]. Compared with their DLCO reference values for adults, the most recent GLI DLCO reference values are notably lower [15]. Another explanation for this difference may consist in the fact that none of the patient in Wu’s cohort have received mechanical ventilation likewise in the cohort of Huang (< 1%) [7, 16]. This may be a message in favor of the implementation of lung protective ventilation in COVID-19 related ARDS. Compared to the population with all-cause ARDS followed by Herridge et al., in whom 3-month DLCO was 63% of predicted (IQR: 54–77) [2], respiratory function recovered more quickly in our cohort of patients with severe SARS-CoV-2.

Although the 6MWT showed significant recovery between 3 and 12 months in most patients, the walked distance remained below the predicted distance in 18 patients (27.3%), and 37.9% of patients had a drop of 4% or more in SpO_2_ at 12 months. Our results are concordant with the cohort of Wu et al. who reported around 14% of patients with 6MWT distance below the predicted value.

SARS-Cov-2 infection may have caused myopathic changes, also represented by the MIP (% of pred) or MEP (% of pred), which showed a constant improvement over the 12 months of follow-up [17].

Creatinine increased at the time of ICU hospitalization was shown as a predictive of low DLCO in the cohort follow-up. The association between creatinine and DLCO can be explained by the high prevalence of thromboembolic events. Reinforcing this hypothesis, the median level of D-dimers was high at the time of hospitalization with a frequency of pulmonary embolisms of 24%. We believe that these patients may have abnormal tiny blood vessels or microthrombus formation, which can be implicated in the reduction of DLCO over time or kidney injury. Other studies have demonstrated a higher rate of thromboembolic events in survivors of COVID-19 at 6 months follow-up, raising the question of the number of undiagnosed cases of segmental or subsegmental pulmonary embolism [18, 19]. The endothelial inflammation classically described in COVID-19 remains a good hypothesis for the increased incidence of pulmonary embolism, and potentially the leading cause of the persistent reduction in DLCO and kidney function over time with a specific increase in D-dimer levels [20]. In our study, D-dimer at the time of hospitalization was not showed to be correlated with KCO measurement at 3 months. Thus, other hypotheses can be put forward on the initial reduction of DLCO with normal KCO apart from microthrombus formation. Considering the number of patients with a history of chronic lung disease and obesity in our cohort, a re-distribution of the regional ventilation/perfusion ratio might have partially compensated the gas transfer efficiency, maintaining satisfactory KCO levels in some patients. Moreover, KCO may have been overestimated due to a functional restrictive pattern [21] that may have masked the possible persistence of damage to the vascular component [22].

At the 12-month CT scan, reticulations were observed in 86% of patients, traction bronchiectasis in 72% and ground glass opacities in 53%. These abnormalities were not significantly associated with DLCO alteration because they only concerned a small percentage of the lung area (< 25%) in most patients. Wu et al. reported abnormal chest imaging in 24% of patients, including interstitial thickening in 5% and reticular opacity in 4%. This higher proportion of persistent CT abnormalities at 1 year could be explained by the greater severity of our patients (85.2% of patients intubated in our cohort *vs* none in the series by Wu et al.).

Our analyses showed a slight-to-moderate deterioration in HRQoL in all domains except for the role-emotional domain. The most altered domains were vitality and role-physical, with a median score of 55 for vitality at 3 months. The only domain that improved over time was the role-physical domain. In an observational cohort by Vlake et al., 118 COVID-19 ICU survivors were evaluated using the same questionnaire [23]. These authors observed that mental HRQOL increased between 6 weeks and 3 months, and remained stable thereafter. Only the role-physical domain improved constantly over time, as in our cohort. A possible explanation might be that for all components except for the physical domain, improvements occur before 3 months, with a ceiling effect already reached by the time the study questionnaires were administered [24]. Compared to the population with all-cause ARDS followed by Herridge et al., HRQoL increased over time for each domain, and particularly the role physical domain, as in our cohort. At the 3-month evaluation, HRQoL scores were lower in the cohort of all-cause ARDS followed by Herridge et al. [2]. These results suggest, as in the paper by Vlake et al. comparing HRQOL results with those of a historical non-COVID-19 post-ICU population, that HRQOL is less deteriorated after an ICU stay for SARS-COV2 than after other causes of ARDS. When we investigated the relation between HRQoL and objective parameters, such as the 6MWT or CT scan findings, there were no significant relations, suggesting a discrepancy between the patient's perception and the functional parameters measured in our study. At 3 months, among the 50 patients (63.3%) who had walk distances below their age-adjusted predicted values, the median (min–max) score in the vitality domain was 55 (5–100), which was the same score as in patients with no alteration on the 6MWT. Vaes et al. reported survey results of post-discharge SARS-CoV-2 patients and they showed significant improvements in work productivity and functional status, although a proportion of patients had persistent symptom, moderate-to-poor health and impaired quality of life, as in our data [25]. No explanation was given for the persistence of an alteration in these domains, and some authors have characterized these patients with the term "long COVID" [26].

The last point to highlight from our cohort is that there were few indicators of psychological disorders, such as depression or anxiety, according to the self-reported HADS score. Using the HADS score at 3 months after hospital discharge, the results reported by Vlake et al. in their cohort of 118 post-ICU patients showed a higher frequency of depression and anxiety [23]. However, in our study, patients had early psychological follow-up and were encouraged to use new technologies to interact with their loved ones, as suggested by Kennedy et al. [27]. It has been reported that survivors of infectious diseases, such as SARS-CoV-2, are exposed to psychological risk, due to contagiousness, extensive isolation measures and public fear of the disease [28]. PTSD would have been interesting to evaluate. Indeed, in the cohort by Vlake et al., 23% of the 57 patients who responded to the IES-R questionnaire had psychological distress, including probable posttraumatic stress disorder (PTSD) in 7% of cases at 6 weeks after hospital discharge.

Our prospective study has some limitations. The severity of the epidemic and the significant mobilization of medical teams made it impossible to consider the constitution of a multicenter cohort at such short notice. Furthermore, some practices have changed based on the lessons learned from the epidemic. For example, the role of dexamethasone in hospitalized patients with COVID-19 has been established [29]. No effects of steroid use, ventilation supports, neuromuscular blocking agents, or prone position on DLCO could be demonstrated but this may be due to the too small size of our cohort. It is recommended to perform contrast-enhanced chest CT scan. Another potential bias is the lack of data regarding the patients’ respiratory function or existence of possible obstructive sleep apnea before contracting SARS-CoV-2. To minimize this potential bias, patients known to have chronic respiratory insufficiency, those on long-term oxygen therapy or those followed for interstitial lung disease were excluded from the cohort.

## Conclusion

In this large cohort of ICU survivors of SARS-CoV-2 infection, systematic multidimensional evaluation up to one year after symptom onset showed that most patients had an improvement in DLCO at 3, 6, and 12 months, and patients who normalized their DLCO did not subsequently deteriorate.

## Supplementary Information

**Additional file 1: Fig. S1.** Flow chart of the COV-RECUP population according to DLCO status. Patient 77 had borderline data at each visit.

## Data Availability

The datasets used and/or analyzed during the current study are available from the corresponding author on reasonable request.

## Abbreviations

ARDS: Acute respiratory distress syndrome
CPET: Cardiopulmonary exercise testing
CT scan: Computed tomography scan
DLCO: Lung diffusing capacity for CO
HADS: Hospital Anxiety and Depression Scale
HRQoL: Health-related quality of life
ICU: Intensive care unit
IQR: Interquartile range
LLN: Lower limit of normal
MEP: Maximal expiratory pressure
MIP: Maximal inspiratory pressure
SARS-CoV-2: Severe acute respiratory syndrome coronavirus 2
SF-36: Medical outcomes study (MOS) Short-Form 36-item questionnaire (SF36)
6MWD: Six-minute walk distance
6MWT: Six-minute walk test
