# Supplementary material for: Result of one-year, prospective follow-up of intensive care unit survivors after SARS-CoV-2 pneumonia
Source: Ann Intensive Care. 2022 Mar 9;12:23. doi: 10.1186/s13613-022-00997-8 (PMC8905558; doi:10.1186/s13613-022-00997-8)
Supplement: Supplementary file 1 — Additional file 1: Fig. S1. Flow chart of the COV-RECUP population according to DLCO status. Patient 77 had borderline data at each visit. [file 13613_2022_997_MOESM1_ESM.docx]

**Figure S1. Flow chart of COV-RECUP population according to DLCO status**

~~
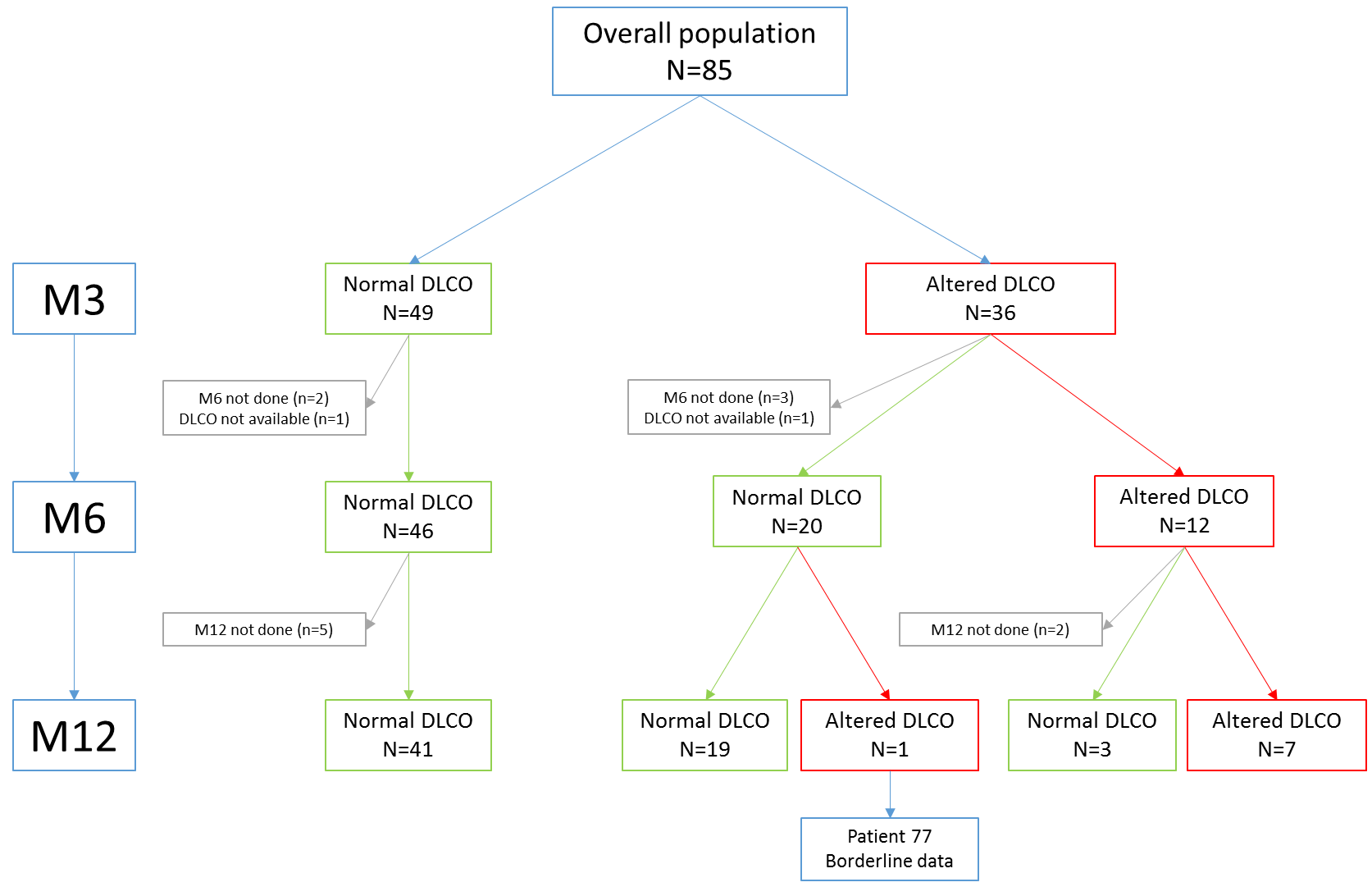
~~

Flow chart of COV-RECUP population according to DLCO status. Patient 77 had borderline data at each visit.
